# An Optimized Relay Selection Technique to Improve the Communication Reliability in Wireless Sensor Networks †

**DOI:** 10.3390/s18103263

**Published:** 2018-09-28

**Authors:** Suelen Laurindo, Ricardo Moraes, Ríad Nassiffe, Carlos Montez, Francisco Vasques

**Affiliations:** 1 Department of Automation and Systems, Federal University of Santa Catarina, Florianópolis 88040-900, Brazil; suelen.m.l@posgrad.ufsc.br (S.L.); carlos.montez@ufsc.br (C.M.); 2 Department of Computer Engineering, Federal University of Santa Catarina, Araranguá 88906-072, Brazil; 3 INEGI, Faculty of Engineering, University of Porto, Porto 4200-465, Portugal; vasques@fe.up.pt; 4 Catarinense Federal Institute, Blumenau 89070-270, Brazil; riad.nassiffe@ifc.edu.br

**Keywords:** relay selection, cooperative diversity, cooperative communication, wireless sensor network, WSN

## Abstract

Wireless Sensor Networks (WSN) are enabler technologies for the implementation of the Internet of Things (IoT) concept. WSNs provide an adequate infrastructure for the last-link communication with smart objects. Nevertheless, the wireless communication medium being inherently unreliable, there is the need to increase its communication reliability. Techniques based on the use of cooperative communication concepts are one of the ways to achieve this target. Within cooperative communication techniques, nodes selected as relays transmit not only their own data, but also cooperate by retransmitting data from other nodes. A fundamental step to improve the communication reliability of WSNs is related to the use of efficient relay selection techniques. This paper proposes a relay selection technique based on multiple criteria to select the smallest number of relay nodes and, at the same time, to ensure an adequate operation of the network. Additionally, two relay updating schemes are also investigated, defining periodic and adaptive updating policies. The simulation results show that both proposed schemes, named Periodic Relay Selection and Adaptive Relay Selection, significantly improve the communication reliability of the network, when compared to other state-of-the-art relay selection schemes.

## 1. Introduction

One of the most pursued goals in today’s computing environments is the ability to run and access computing data, anywhere, anytime. This concept—commonly known as ubiquitous computing—can be achieved using the emergent Internet of Things (IoT) paradigm, where sensors and actuators are seamlessly integrated into the environment [1]. One of the major objectives of IoT is to enable smart objects or “things” to communicate with each other and cooperate to achieve a common objective [2,3]. Due to their characteristics, such as low cost, ease of deployment, ability to closely monitor physical phenomena of interest, Wireless Sensor Networks (WSNs) provide a suitable support for low-rate monitoring applications, and are considered as one of the enabling technologies for the dissemination of the IoT paradigm [2,4].

Nevertheless, WSNs are subject to restrictions in what concerns the exhaustion of energy resources and the unreliability of message communication [5,6]. Energy consumption is a problem because nodes have limited energy resources. Normally, they are powered by chemical batteries, that when discharged must be either replaced or recharged. However, it is not always easy to replace batteries, especially in large-scale networks. To reduce the energy consumption of WSN nodes, these type of networks are usually configured to follow a duty-cycle operation. That is, nodes will periodically switch off parts of their circuits, becoming idle during significant periods of time. Moreover, low communication reliability may lead to a severe reduction of the achievable throughput in WSNs. This problem is associated with messages being lost due to electromagnetic noise and/or other devices that operate in the same frequency range or obstacles between nodes [7].

A possible solution to improve the reliability of WSNs is by providing multiple paths to transmit data from the source to the destination node. As a consequence, a message transfer would still be possible if there are other available links whenever a link fails. This type of communication is referred to as cooperative diversity, and it allows the enhancement of communication over unreliable channels [8].

In cooperative diversity techniques, networks may use single-antenna equipments to achieve the same benefits of multiple-input-multiple-output (MIMO) systems. It allows a set of nodes to exploit their antennas to form a virtual MIMO system, avoiding the deployment of multiple antennas that would increase the overall energy consumption [9]. Unlike traditional WSN communication, where packets have a destination address and the transmission involves only one transmitter and one receiver, cooperative diversity considers the existence of nodes that will cooperate with the transmitter–receiver pair, listening and storing messages received from their neighbors, and then retransmitting them to the destination node. Thus, the destination node has a higher probability of receiving the sent packets because messages that were not directly received by the destination node may be received during the retransmission phase [10]. This communication behavior allows a better usage of the broadcast nature of wireless transmissions [11], since the message diffusion can be heard by the neighboring nodes (as long as they have their radios on), improving the network success rate without increasing its hardware complexity.

Whenever using cooperative diversity, the selection of the set of relays is of paramount importance to achieve a good performance level. It is important to point out that selecting all nodes as relays would allow a greater cooperative diversity. However, at the same time, it would increase the number of message collisions, the overall energy consumption, and would affect the synchronization among all those nodes [9]. Consequently, one of the major challenges of cooperative retransmission techniques is the selection of the best set of relay nodes, in order to improve the overall communication reliability [12].

The selection criterion is one of the most relevant aspects when selecting the set of relays nodes. When selecting the cooperator nodes, many studies consider just quality estimators for this purpose, such as Received Signal Strength Indicator (RSSI), Channel State Information (CSI), Signal-to-noise ratio (SNR) or Link Quality Indicator (LQI) [7,13,14,15,16,17]. However, the consideration of just this type of quality estimators may generate inaccurate decisions [18]. In fact, hardware metrics such as LQI, RSSI and SNR are based on just the first eight symbols of a received packet, and are therefore unable to provide an accurate estimate of the link quality. More importantly, they are only measured for successfully received packets. As a consequence, whenever there are frequent packet losses, the above metrics will overestimate the quality of the link. Therefore, despite providing a quick way to classify communication links as good or bad, this type of link quality estimators are unable to provide accurate estimates. Thus, they should not be independently considered, but rather by combining them with other metrics [18].

**Figure 1:**
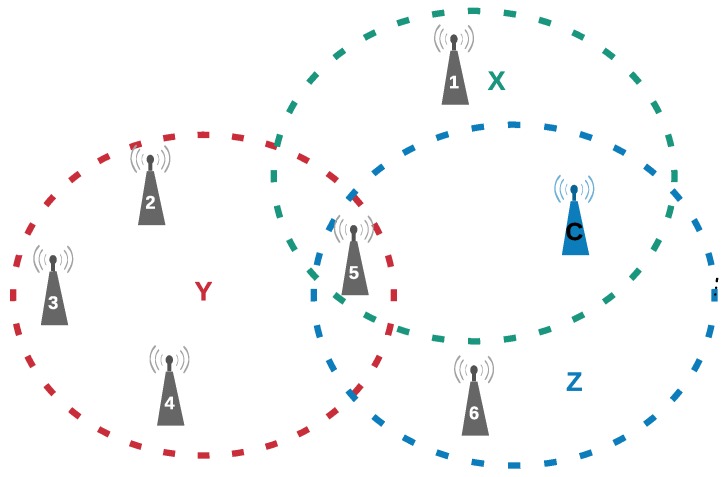
Wireless sensor network that needs a relay.

Figure 1 presents a WSN composed of seven nodes, one being the coordinator node. In this WSN, there are three regions identified as X, Y and Z. All nodes in the same region are considered to be neighbors and can hear each other. Nodes 2, 3 and 4 that are in the Y region are not able to directly communicate with the coordinator. In this network, it is possible to visualize the need for using relays, so that messages from all nodes of the network can reach the coordinator node. In addition, it is also possible to observe that to select adequate relays it would be necessary to consider other parameters besides the channel quality. For instance, nodes 1 and 6 have a good communication link with the coordinator, but, they are far from the nodes that need to use cooperation techniques to reach the coordinator. In this scenario, it would be more appropriate to select node 5 as the relay node.

On the other hand, whatever the parameters used to perform the relay selection, the updating activity can follow a periodic or an adaptive strategy [16]. In the periodic strategy, the relay updating always occurs, independently of the network requirements. An adaptive strategy considers a specific updating policy, which considers relevant modifications of the environmental conditions to trigger a new relay selection.

This paper proposes a new relay selection technique to be used in WSNs, named Optimized Relay Selection Technique (ORST). It also investigates the use of two different relay updating schemes. The term optimized is used in this paper in the sense of an improved way to select relay nodes, when compared to other state-of-the-art approaches. The periodic scheme, named Periodic Relay Selection (PRS), is an extension of the scheme proposed in [19], where the relay selection is periodically triggered, without analyzing the need for a new selection. In the adaptive approach, named Adaptive Relay Selection (ARS), the time interval between consecutive relay selections is dynamically determined, according to the network’s success rate. The ARS scheme is able to handle dynamic networks, where nodes may randomly join or leave the coverage area of the coordinator node.

For both relay selection schemes, the aim is to maximize the communication success rate and also to minimize the energy consumption of the network, by selecting the smallest number of relay nodes and, at same time, ensuring that all nodes are linked to at least one corresponding relay node. When compared to other techniques, the main scientific contribution of the proposed technique is the selection of a set of relay nodes being based on multiple criteria, namely: the number of neighbors of the candidate node, their remaining battery energy, the quality of the communication link between the candidate node and its neighbor nodes (by using the RSSI) and the success rate’s history in recent node transmissions, which provides the adequate selection of relay nodes and improve the communication reliability, since these criteria are critical for the operation of the network. However, this technique is more complex than when using just a single criterion to rank the candidate nodes to become relays. As multiple constraints are included in the relay selection model, the proposed scheme is formulated as an optimization problem, using a specifically selected benefit function.

A simulation assessment of both schemes was performed using the OMNeT++ tool [20]. The proposed approaches were compared with three state-of-the-art techniques: Opportunistic [7], which selects the cooperating nodes according to the network packet error rate, Random Around the Coordinator [21], which performs a random selection of the nodes that have an adequate communication link with the destination node and Completely Random relay selection [22], which performs a random selection from all the nodes of the network.

This paper is structured as follows: Section 2 presents the state-of-the-art in what concerns relay selection techniques in WSNs. Section 3 describes the proposed relay selection technique, which aims at the improvement of the communication reliability in WSNs. Section 4 presents the simulation assessment of the proposed approach. Finally, conclusions are presented in Section 5.

## 2. Related Work

**Figure 2:**
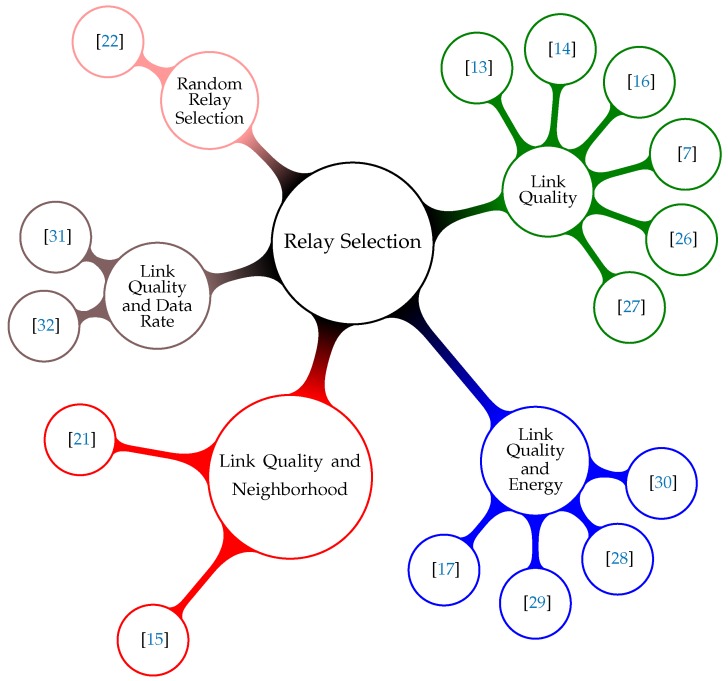
Relay selection categories based on the criteria used to select the cooperating nodes.

The adequate selection of relay nodes is fundamental to improve the performance of cooperative communication techniques [21]. There are multiple schemes to select relay nodes in the literature. However, many of these works usually focus on the analysis of the data communication protocols, evaluating and proposing different techniques for sending messages (or signals) by the relay nodes, based on Amplify-Forward (AF), Decode-Forward (DF) or Hybrid approaches [23,24,25]. As a limitation of these works, it can be mentioned that they do not deal with the selection of retransmission nodes, or, when they do, they assume only a single criterion for such a selection. This work aims to improve the relay selection in WSNs. In this way, the objective of this section is to describe state-of-the-art approaches that mainly deal with the relay selection procedures. Then, state-of-the-art selection schemes were classified into five categories, according to the criteria used for the relay selection: Link Quality Based  [7,13,14,16,26,27], Link Quality and Energy Based  [17,28,29,30], Link Quality and Neighborhood Based  [15,21], Link Quality and Data Rate Based  [31,32] and Random Relay Selection  [22] (Figure 2).

### 2.1. Link Quality Based

In this category, the relay selection only considers the use of a link quality estimator. Liu et al. [26] proposed a relay selection scheme for clustered wireless networks. The authors considered that there should be a relay set in the source node’s cluster (transmitter *T*) and a relay set in the destination node’s cluster (receiver *R*), with a maximum number of relays *K*, such that, |*T*| + |*R*| ≤ *K*.

The set *T* is incrementally formed, selecting relays with the maximum channel gain up to the relays of set *R*. The selection occurs until the number of relays reaches *K* or until the SNR requested by relay in *R* is reached. To select the receiver set *R*, the authors defined that only nodes with a non-negative SNR margin could be qualified as relay receivers (QR). Two heuristics were proposed to further reduce the searching space of *R*. The basic idea is to define a set of candidate receivers (CR) based on QR. In the first heuristic, the selected relays are the nodes with more capability to decode the received signal from the transmitting relays. In the second heuristic, the selected relays are the nodes geographically closer to the destination node, which present the maximum achievable SNR margin up to the destination node.

After defining the CR set, the receiver relay set *R* is selected considering just the nodes belonging to CR. The nodes in CR are arranged in decreasing order according to the SNR to the destination node and the set CR will be the input to determine the transmitting relays set *T*. If a feasible solution can be found, then both sets CR and *T* are the optimal solutions.

Marchenko et al. [16] presented an adaptive relay selection based on LQI, where the relay selection is triggered depending on the recent delivery ratio performance of the cooperative link. A source node *S* monitors the acknowledgments (ACK) of the sent packets. If the ACK for a packet is missing, it assumes that neither *S* nor the currently assigned relay $R_{i}$ could deliver this packet. If the ratio of missing ACKs during a contention window $W_{a}$ is equal or higher than a threshold value $\varepsilon_{a}$, a new relay selection is triggered. To perform the selection of relay nodes, *S* broadcasts a relay request message, which also includes the identification of the destination node *D* for the following data packets. All nodes receiving this request will trigger a random timer $T_{c}$ = rand(0,*W*) for a transmission during a contention window of duration *W*. When the timer of a node expires, it sends a candidature message to *D*. This message includes the measured LQI value and the value of *W*-$T_{c}$. Thus, *D* can identify the end of the contention window even if it has not received the relay request message. Nodes whose candidature message is received at *D* will form a relay candidate set *R*. When the contention interval ends, *D* evaluates the end-to-end link quality for each candidate node $R_{i}$ by taking the minimum of two LQI values (*S* to $R_{i}$ and $R_{i}$ to *D*).

Li et al. [13] proposed a relay selection technique using CSI as criterion, which can be performed either as a centralized or a decentralized approach. In the centralized approach, the relay selection is done at the destination node. In the decentralized approach, nodes estimate their channel gains through the exchange of handshake messages Request-To-Send (RTS) and Clear-To-Send (CTS) and trigger a timer with a value inversely proportional to the gain. The node that has the first expired timer is selected as relay.

Ferdouse and Anpalagan [14] proposed a relay selection scheme based on the use of the Bayes’s theorem. The authors consider CSI and SNR values between source-destination pairs and the source-relay pairs as criteria for the selection. The authors use two factors for the relay selection. The first performs the relay selection, the destination node or the source node; and the second is responsible to define the retransmission technique, AF (Amplify-and-Forward) and DF (Decode-and-Forward). The authors also assume that the CSI value, the achievable data rate under AF ($C_{AF}$), and the data rate in direct transmission ($C_{D}$) are known. Based on the CSI information, it is calculated the $SNR_{D}$, the $SNR_{AF}$ or the $SNR_{DF}$. Also based on the SNR value, the capacity of the channel from the source node to the destination and from the source node to the possible relays is calculated. Afterwards, it is calculated the prior probability for each source node $P\left(S_{1}\right)\ldots P\left(S_{n}\right)$ and the conditional probability for each possible relay $P\left(R_{1}|S_{1}\right)\ldots P\left(R_{n}|S_{n}\right)$, applying the Bayesian theorem to determine the posterior probability for each source node and possible relays in the network $P\left(S_{1}|R_{1}\right)\ldots P\left(S_{n}|R_{n}\right)$. The relay selection is based on the maximum posterior probability, if $P\left(S_{n}|R_{k}\right)>P\left(S_{n}|R_{j}\right)$ to k≠j, then the $R_{k}$ will be selected.

The main difference between the source and destination methods is that when a source node performs the relay selection, it collects information about the channel of the possible relay during the beacon period, measures its SNR and estimates the posterior probability, while the destination node collects all channel information between source-relay (CSI and SNR) to calculate the posterior probability and assign a relay to all active transmission (source and destination) pairs.

Andre et al. [27] presented an adaptive and decentralized relay selection technique, where the relay selection occurs each five failed transmissions. The criteria used for the relay selection is the LQI, where a timer inversely proportional to the link quality is triggered at each node. Thus, the node with the best quality will have its timer expired first and will be the relay node. The operation takes place as follows: the source node broadcasts a relay frame; potential relay nodes that listen to this message will store the channel quality with the source node and wait for the response of the destination node. When the destination node responds, the nodes that listen to the message store the channel quality to the destination. Only the nodes that listen to both messages may dispute the relay selection. The candidate relays will activate a timer inversely proportional to the link quality from the source node to the destination node. When the timer expires, the candidate relay sends a message to the source node to inform that it will be the relay and, after receiving it, the source node sends a message signalling that the relay has already been selected. Thus, if after a source node sending a message, the destination node does not send its ACK, which means that the relay node will retransmit the message.

The authors conclude that the adaptive relay selection is beneficial compared to the periodic selection, improving the delivery rates. However, the improved reliability comes at the cost of an increased overhead, as this technique requires additional message exchanges between the source and the destination nodes. Additionally, as the adaptive relay selection is more frequently triggered than the periodic technique, it also generates an increased overhead.

Another relay selection technique, called Opportunist, was proposed by Valle et al. [7]. This technique considers the history of successful transmission rates and the LQI between each node and the coordinator. To select the best relay nodes among the candidates, the authors propose the following equation:

(1) $$CN_{i}=\frac{SR_{i}+LQI_{i}}{2},$$

where, for each node *i*, $SR_{i}$ is the history of recent successful transmission rates and $LQI_{i}$ is the quality indicator of the link between the node and the coordinator.

Nodes presenting the highest $CN_{i}$ will be selected as relays. The number of relay nodes is dynamically defined according to the percentage of message losses in the network. The coordinator notifies the relay nodes through a special message called *Blocop*. The selected relays store all messages that are sent by all nodes during each of the T slots (where T is the total number of slots within the transmission period). After the end of the T slots, the relay nodes encode the set of messages stored in their buffers and wait until specific slots to perform the retransmissions.

### 2.2. Link Quality and Neighborhood Based

In this category, Etezadi et al. [21] proposed three techniques for the selection of relay nodes. The first one uses the source-to-destination SNR as criterion; the second is based in a geometrical analysis, selecting nodes closer to the source node; and the third one randomly selects relay nodes located in an *R* radius around the coordinator.

The first two techniques have a higher energy consumption. In the first, the authors assume that SNR can vary frequently. Thus, the set of nodes with the highest SNR can be modified between two consecutive transmissions. This fact suggests that, in this scheme, all nodes are possible candidates to be relays, and, therefore, should always switch between the listening and the transmission mode without the privilege of going to sleep and preserving their energy. In the second, the set of nodes closest to the source node rarely undergo updates. Therefore, these nodes continue to switch between the listening and the transmission modes, leading to an early exhaustion of their batteries, while all the other nodes are able to remain in sleep mode and saving energy. The third technique randomly selects a new group of relay nodes among the nodes that are within a radius *R* around the source node O(S,R). According to the authors, this technique is the one with the lowest energy consumption.

Alkhayyat, Gazi and Sadkhan [15] presented a relay selection scheme whose aim is to select the best relay node considering the quality of the possible relay with the destination node in consideration. The authors defined a selection area and only the nodes that are within this area can participate in the relay selection. This area is defined as follows: when a source node has a message to transmit, it begins by transmitting an RTS message to the destination. If the destination receives the message correctly, it will transmit a CTS message to the source node. The nodes that listen to both messages (RTS/CTS) are considered to be within the selection area and can dispute the relay selection.

After the RTS/CTS message exchange, the source node will transmit its message, and the nodes that are both within the selection area and are able to decode the message will dispute the relay selection. Each of these nodes will initiate a contention period, during which a backoff time function decreases based on the distance and the received signal quality from the relay to the destination node. The evaluation of this function is done at each node, based on the information obtained through the handshake messages (RTS/CTS). Thus, the node that gets the lowest value from the backoff time function will access the channel first and will be the relay.

### 2.3. Link Quality and Energy Based

In this category, Brante et al. [28] presented a decentralized fuzzy-based relay selection technique, that uses the relay-to-destination CSI and the node’s battery level ($E_{i}$) as parameters. It considers that each node is able to read its battery level($E_{i}$) and to estimate the state of its channel ($g_{iD}$) using the NACK message sent by the destination node. Each node evaluates its degree of relevance to act as relay, which is a function of $g_{iD}$ and $E_{i}$. The higher *f*($g_{iD}$,$E_{i}$) is, the higher is the quality of the relay. Thus, when the node gets its degree of relevance, it waits for a time interval $T_{i}$ to avoid a collision, in case more than one node presents the same degree of relevance. In the sequence, it will act as a relay and retransmit the message to the destination node.

Ahmed, Razzaque and Hong [29] proposed a relay selection protocol that aims to maximize the network lifetime and to minimize the end-to-end delay in packet delivery. The authors consider that each node is able to evaluate a weighted average *W* from its residual energy and the average delay of sent packets. The protocol operation is as follows: the source node sends an RTS message and the destination node responds with a Request-To-Help (RTH) message containing WH and WL values, which are defined by the destination node and correspond to a highest and lowest threshold *W*. Nodes that listen to both messages (RTS/RTH) will evaluate their weight *W* and will contend to send a message Interested-to-Help (ITH) if its *W* value satisfies the condition $WL\leq W_{n}\leq WH$.

In this technique, if more than one node has a *W* value that satisfies the condition $WL\leq W_{n}\leq WH$ a collision may occur, which will make the relay selection process slower. Whenever a collision occurs, the destination node will increase the value of its lower threshold WL, aiming to reduce the number of nodes that can be within the range of WL and WH. Then, nodes that still satisfy the constraint try to send the ITH message again. The first node that is able to send its own message to their destinations will be the relay.

Pham and Kim [17] presented a relay selection technique that aims to increase the network lifetime and improve its packet delivery. The node with the highest residual energy level and the lowest Energy-Per-Bit value (EPB) will be the relay node. The basic idea is that, based on the CSI and residual energy information (REI) of each relay, it first decides which protocol is better for the transmission. If the channel SNR between the source node and the cooperating candidate node is greater than a previously defined value, the node will use AF, otherwise, it will use DF. After selecting a suitable forwarding protocol, the node with the higher residual energy $e_{i}^{m}$ and the lower EPB (*Energy-Per-Bit*) $E_{b}$ will be selected as relay. Thus, the authors proposed the calculation of a weight *w* for each cooperating node *r*, as: $w_{r}=\frac{e_{i}^{m}}{E_{b}}$, where, the relay with the highest weight value is selected.

Cheikh, Simpson and Sun [30] proposed a relay selection technique that aims to decrease the energy consumption of the network. The authors propose an optimization problem that determines which node minimizes the energy consumption per bit in transmissions between source and relay nodes and between relay and destination nodes. The destination node implements an optimization algorithm and selects the node that minimizes the energy consumption.

The operation of this technique is as follows: the source node sends an RTS message, signaling that it has data to transmit; the destination node responds with a CTS message. The nodes that listen to both messages estimate the channel gain and the desired transmission power to reach the target BER. Using an optimization strategy, the destination node selects the relay node, and signals through the beacon message which was the selected node.

### 2.4. Link Quality and Data Rate Based

In this category, Gokturk and Gurbuz [31] proposed two techniques for the selection of relay nodes, using Frame Error Rate (FER) as selection criterion, one being centralized and the other decentralized. In the centralized technique, the source node will incrementally select the nodes in the relay set. The number of relays in the set is incremented until the set satisfies the target FER, as long as the set presents a minimum power consumption. The nodes that were able to successfully decode messages coming from the source nodes are part of the relay set. In the decentralized technique, each node decides whether it will be relay or not and calculates its own optimal power level. Each node that was able to decode the message from the source node calculates the power allocation required to satisfy the target FER in the transmission. If a node concludes that its inclusion in the relay set can help the achievement of the required FER, it announces its intention for cooperation. Each time that a node advertises its availability to cooperate, the other nodes calculate the optimal power assignment and the energy cost for the new relay set and compares it with the energy cost of the set before its insertion. The algorithm continues until the energy cost per bit can not decrease any more. If no nodes are available for the cooperation or the FER requirement can not be satisfied with the cooperation of the available nodes, the cooperation is aborted.

Ouyang et al. [32] presented a blind relay selection technique, based in a random medium access mechanism (ALOHA). The idea is that the node able to achieve a required transmission rate at the destination and able to access the channel first will be the relay. Due to high collision probability, the authors determine that only the nodes that are within a given region called ARR (Available Relay Selection) will participate in the selection. To be in this region, the nodes need to satisfy a required transmission rate. The operation of the proposed technique occurs in three phases. In the first phase, the target is to identify the possible relay nodes. Thus, the source nodes transmit a message with the required rate $R_{r}$ and their locations. The nodes that listen these messages will evaluate which nodes are able to achieve the required rate. The nodes that satisfy the $R_{r}$ rate respond with a “ready-to-relay” message. In the second phase, all possible relays will start a competition phase based on a slotted ALOHA medium access so that the relay selection may occur. Each possible relay transmits a “select-me” message to the source node; if just one node transmits a message within the slot, it means a successful selection and the relay is selected for the retransmission in the current frame. Otherwise, a collision occurs and the nodes will attempt to send the message in the next slot. Phase two ends whenever a selection occurs (that is, when the first “select-me” message sent by a possible relay successfully reaches the source node). When a selection occurs, phase three begins, starting the retransmission.

### 2.5. Random Relay Selection

Finally, in this category, the relay selection is performed without considering any criteria; therefore, any node in the network can be selected as relay. Willig and Uhlemann [22] conducted a study about this topic and concluded that relay nodes do not need to be in specific positions, which often requires specific channel quality measurements. According to the authors, randomly selected relay nodes can also provide benefits to a WSN, improving its success rate.

### 2.6. Wrap-Up

Table 1 summarizes the described works, comparing them among themselves with respect to the following set of classifiers: used category to select the relays, if the selection technique uses a periodic (P) or adaptive (A) approach and if it requires the use of additional message exchanges.

From Table 1, it is possible to highlight some common characteristics. For instance, a large number of works consider just the link quality factor, which may generate bad decisions when selecting the set of relays [18]. The main reason is that hardware metrics are based on the sample of just the first eight symbols of a correctly received packet, not considering the lost packets. Thus, it may provide an inaccurate estimate of the link quality.

Another characteristic that can be observed is that most of the proposed techniques require the use of additional message exchanges to make the selection. Many of them use handshake techniques (RTS/CTS) to estimate the channel quality between source-relay-destination nodes. However, when it comes to WSNs, handshake messages may generate a relevant overhead in the network and, in case of collisions, may generate considerable additional delays.

Finally, most of the presented works periodically perform the relay selection. A periodical selection may be inefficient for two reasons. First, the relay selection can be unnecessarily triggered because the current relay remains the best option. The second reason is that, if the selection always occurs at a fixed interval, there is always the possibility of an outdated relay selection to occur. For instance, a node may leave the network, a new node may join the network or even an increase in the level interference may occur. If the selection periodicity is long, there is a risk that the current set of relays may not be the best one. Thus, whenever the network has a dynamic behavior, it is necessary to avoid these problems using, for instance, adaptive selection techniques.

To overcome some of the disadvantages highlighted by this state-of-the-art analysis, the technique proposed in this paper considers multiple criteria as parameters for the relay selection. In addition, it does not require the exchange of any additional messages to converge.

## 3. ORST-Optimized Relay Selection Technique

In this paper, it is proposed a centralized relay selection technique, named ORST (Optimized Relay Selection Technique). The selection of relay nodes is formulated as an optimization problem, using a benefit function that considers the following selection parameters: (a) the number of neighbors of the candidate node; (b) its remaining battery energy; (c) the quality of the link between the candidate node and its neighbor nodes; and (d) the history success rate in recent node transmissions. In addition, this paper also investigates the usage of two different relay update schemes, PRS (Periodic Relay Selection) and ARS (Adaptive Relay Selection).

### 3.1. System Model

The system model considers a network organized in a star topology. It is also considered that nodes without a direct link to the coordinator will use a neighbor relay to establish a communication link with the coordinator. The use of a star topology in industrial applications is commonly justified due to its advantages in terms of latency, synchronization, simplicity and also due to its energy efficient behavior [7,33].

A slotted communication approach is assumed, where the medium access is based on a TDMA scheme. This type of communication approach is of common use whenever dependable communication is required, as it increases the communication reliability allowing the medium access without contention [5,34].

The IEEE 802.15.4 standard operating in beacon-enabled and time-slotted mode is adopted for the PHY (Physical) and MAC (Medium Access Control) layers of the network. It is important to note that this communication scheme is similar to LLDN (Low Latency Deterministic Networks), which was also used in the cooperative retransmission schemes proposed and assessed in [35].

The proposed relay selection technique assumes that there is specific information exchanged among the coordinator and other nodes during each period of the network, being the information sent by the coordinator piggybacked with the beacon frame and the information sent by the nodes piggybacked with the monitored data.

**Figure 3:**
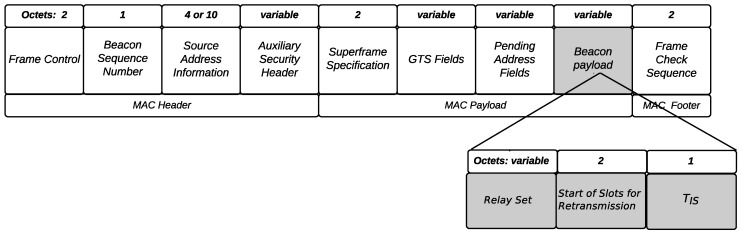
Beacon frame format.

The payload field of the beacon frame is used to send three parameters, Relay Set, which is the information with the identifiers of the selected relay nodes, Start of Slots for Retransmission, which reveals which is the first slot for retransmission (retransmission slots are contiguous slots that are allocated after the transmission slots) and $T_{IS}$, which reveals if the nodes should listen to their neighbors in this beacon interval (BI) or not. Figure 3 illustrates the beacon frame structure.

**Figure 4:**
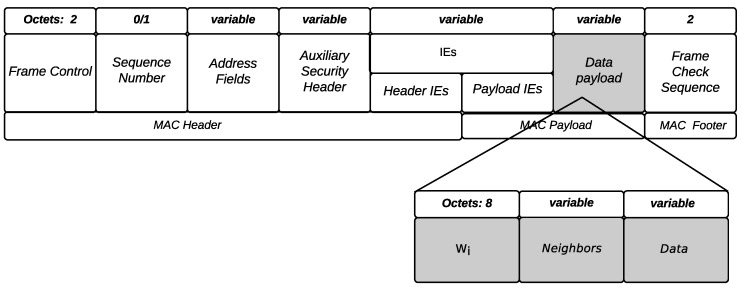
Data frame format.

The data payload field is also used by nodes to piggyback two types of information for the coordinator: Neighbors, which reveals the list of neighbors of each particular node and $W_{i}$ that reveals its benefit value (described in Section 3.3). Figure 4 illustrates the frame structure of a data message.

In the relay message, the payload data field is used to send the list of heard messages. This field is formed by a list that contains just the *id* of the transmitting node and the *id* of the transmitted message, enabling the coordinator to assess the message success rate. As the major focus of the proposed ORST is the adequate selection of relay nodes, no data fusion technique [36], nor retransmission protocols as AF, hybrid approaches or network coding techniques have been implemented [7,23,37,38].

### 3.2. Brief Explanation of the Relay Selection Operation

When a node joins the network, it synchronizes itself with the beacon message sent at the beginning of the beacon interval (BI). Considering a time-slotted medium access, all nodes will make a transmission attempt during their respective time slot. During the first BIs, the network still does not have any relay. After starting the network operation, there will be a configuration period within which nodes will identify their neighbors, calculate the benefit function value (described later), and send this information back to the coordinator node. Only the nodes with RSSI greater than or equal to -87 dBm will be added as neighbors, as this value indicates that there is an adequate communication link between them, as suggested by [39].

During the following BIs, each node is already aware of its neighbors, this information being used to evaluate the benefit value for the relay selection. Afterwards, each node sends to the coordinator its benefit value and the related neighborhood information piggybacked with the data message. The coordinator will use this information to perform the relay node selection. The information about which nodes were selected as relays will then be piggybacked by the coordinator in the next beacon.

**Figure 5:**
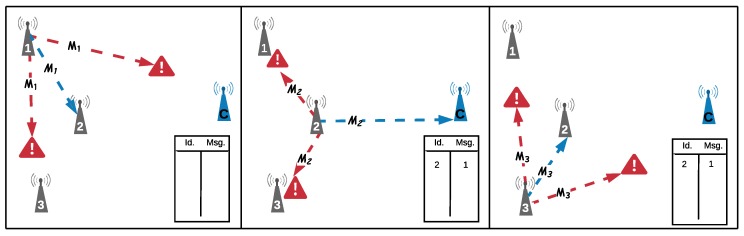
Transmission steps.

After notifying all the relay nodes, the communication will occur in two steps: transmission and retransmission. In the first step, as illustrated in Figure 5, each node make a transmission attempt, the set of relay nodes will stand by listening to and storing all messages sent by all nodes, storing both the successfully received messages and the identification (*id*) of the sending node. If the node is not a relay, it will enter into a sleep mode when finishing the transmission step ($Node_{1}$ and $Node_{3}$ in Figure 5).

After the first selection of relay nodes, the interval between selections will be defined according to the used selection scheme (PRS or ARS). Therefore, this interval can be fixed (PRS) or adaptive (ARS). During the two BIs that precede a new relay selection, all nodes of the network will remain active until the end of the transmission step, as they need to listen to the nearby nodes to update their list of neighbors.

**Figure 6:**
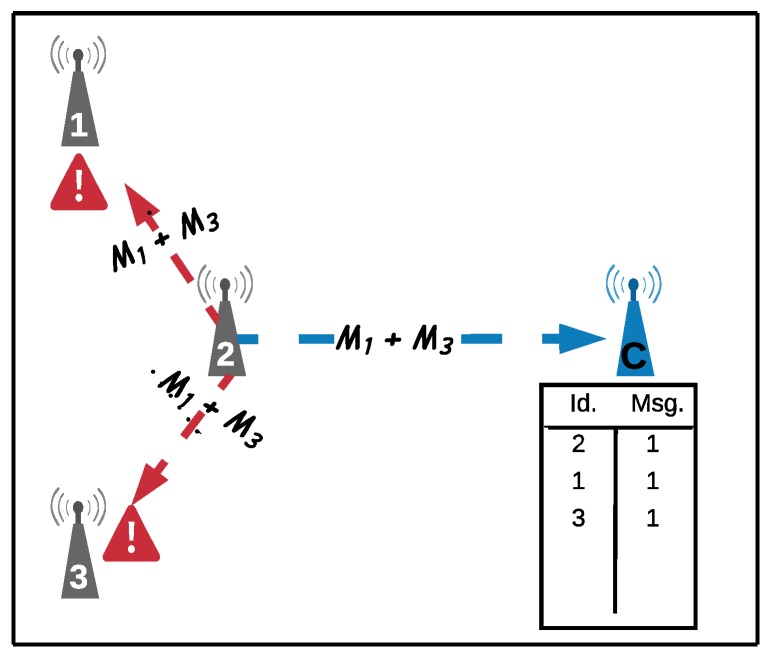
Retransmission steps.

In the second step, represented in Figure 6, each relay node will retransmit one message containing all the *Ids* of the successfully received messages to the coordinator. The coordinator stores in a table all incoming messages and compares them with the received relay messages. Whenever the coordinator receives a retransmitted message that it had previously received, it counts this message as a duplicate, which is a useless message. This information is not used by the proposed selection technique; it will be used just as statistical information. The percentage of received duplicates will be used as a metric to evaluate the efficiency of the relay node selection technique. After the retransmission, the relay will enter in sleep mode.

### 3.3. Problem Formulation

The proposed ORST scheme aims to find a set of relay nodes $S^{⋆}=\left\{y_{1},y_{2},\ldots ,y_{m}\right\}$ among a set of nodes $X=\{x_{1},x_{2},\ldots ,x_{n}\}$ in WSNs, ensuring two conditions: (1) each node $x_{i}$ (1≤i≤n) is covered by at least one relay node; (2) the sum of the weights of the relays is minimized. In this scheme, $x_{i}$ is used as node identifier, *n* is the total number of nodes in the network, *m* is the total number of relay nodes and $S^{⋆}\subseteq X$, i.e., relays are selected in the same set of nodes, transmitting not only their own data, but also cooperate by retransmitting data from other nodes. There is one node called a coordinator in the WSN (*C*).

A node $x_{i}$ will be a candidate to be a relay if and only if: (a) it is neighbor of the coordinator; and (b) it has at least one more neighbor. The ORST scheme is a kind of resource allocation algorithm that may be reduced to the classic set-covering problem applied to WSNs [40].

Then, considering a WSN composed of a set of nodes $X=\{x_{1},x_{2},\ldots ,x_{n}\}$, being that every node has an associate positive weight value and a specific communication range, we construct an undirected and weighted graph G=(X,E) in the following way. Each node $x_{i}$ corresponds to a vertex $x_{i}\in X$ and two vertices $x_{i}$ and $x_{j}$ have an edge $e_{i,j}\in E$ if $x_{i}$ is able to hear a message sent by $x_{j}$ with the value of RSSI ≥-87 dBm, as defined by Srinivasan and Levis [39] as the minimum value for an adequate communication in WSNs.

Every graph with *X* and *E* has subsets $F=\{S_{1},\ldots ,S_{K}\}$, where each subset $S_{k}$ is known as a set cover of the graph *G*. The set-covering problem consists of a finite set *X* and a family *F* of subsets of *X*, such that every vertex of *X* belongs to at least one subset in *F*. Each subset of *F* is formed by vertices that accomplish conditions (a) and (b).

It is said that a subset S∈F covers its element. The problem is to find the set with the minimum sum of weight subset $S^{⋆}\subseteq F$ whose members cover all vertices of *X*.

The WSN problem treated in this paper, which consists of finding the set-cover with minimum sum of weights, is a special case of the set-covering problem. The corresponding decision problem generalizes the well-known NP-complete vertex-cover problem and is therefore also NP-hard  [41,42].

Differently from other relay selection techniques, ORST selection methodology is based on multiple criteria, represented by a weight $W_{i}$ that is assigned for each node $x_{i}$. This weight takes into consideration the available energy in the nodes, the number of neighbors that each node can hear (RSSI ≥-87 dBm), the quality of communication between the source node and the candidate relay node, as well as the history of successful transmission rates of node $x_{i}$. These parameters were selected because they are highly relevant for the operation of the network. For instance, the residual energy of the nodes is an important parameter, considering that, if a node has a low battery level it will stop being a promising candidate, because soon it will exhaust its own energy resources. The number of neighbors that each node has is also a parameter that must be considered, since if a node does not have neighbors, it does not make sense to select it as a relay. The quality of the channel between the source and the relay nodes is another important parameter because it allows for knowing if there is a good communication link between these nodes, ensuring that the relay node correctly receives the message to be retransmitted. Finally, the history of successful transmission rates is an indication that the selected node has a good communication link with the destination node, ensuring that messages sent by this node will correctly arrive to their destination. Combining these parameters as the selection criterion, the target is to ensure that appropriate nodes will be selected as relay nodes. Each node $x_{i}$ will evaluate its benefit $W_{i}$ as follows:

(2) $$W_{i}\overset{∴}{=}\left(\frac{\beta^{v}}{v_{i}}+\frac{\beta^{e}}{e_{i}}+\frac{\beta^{s}}{s_{i}}+\frac{\beta^{H}}{H_{i}}\right),$$

where:

- $W_{i}$ is the benefit value of node $x_{i}$;
- $v_{i}$ is the total number of neighbors of node $x_{i}$;
- $e_{i}=\frac{RE_{i}}{IE_{i}}$, where $RE_{i}$ is the remaining energy and $IE_{i}$ is the initial energy of node $x_{i}$, respectively. The $e_{i}$ value is the normalized remaining energy of node $x_{i}$ (an integer value between 0 and 1);
- $s_{i}=\frac{1}{Limited\_RSSI}\sum_{j=1}^{n_{i}}RSSI_{j}$, where $RSSI_{j}$ is the Received Signal Strength Indicator (RSSI) among node $x_{i}$ and its neighbors nodes $x_{j}$, and the constant limited_RSSI is the minimum value of RSSI for an adequate communication (-87 dBm [39]).
- $H_{i}=\left(1-\alpha \right)\times H_{i}+\alpha \times S_{R}$ is the history of successful transmission rates adjusted at each beacon interval. The value of variable α is adjusted according to each case, being defined between 0<α≤1; variable $S_{R}$ is equal to 1 in case of a successful transmission of node $x_{i}$ or 0 otherwise;
- $\beta^{n},\beta^{e},\beta^{s},\beta^{H}$ are the weights of each parameter for the objective function.

The selection of the set of relay nodes is based on the gain ($W_{i}$) provided by a node $x_{i}\left(i=1,2,\cdots ,n\right)$ up to its set of neighbors $x_{j}\left(j=1,2,\cdots ,v_{i}\right)$. In order to select the minimum number of relay nodes, ensuring at the same time that every node has a reachable relay, an optimization problem is formulated as follows:

(3a)minimize ∑i=1nWiyi,(3b)subject to: Ay≥b,(3c)Cy=d,yi∈{0,1}.

In the constraint presented in Equation (3b), A is the adjacency matrix of order n×n, where its element $a_{i,j}=1$ if node $x_{i}$ is a neighbor of node $x_{j}$ and $a_{i,j}=0$, otherwise. Matrix A is formed on the coordinator node based on the list of neighbors sent by each node of the network. Therefore, whenever the list of neighbors of a node $x_{j}$ has not been received by the coordinator, all elements of row *j* of matrix A will be equal to zero; *y* is a vector of order *n*x1, where $y_{i}$ will be equal to 1 when node $x_{i}$ is selected as relay and 0 otherwise; and *b* is a vector whose $b_{i}$ value has been defined as 1, representing the minimum number of relay nodes of each node $x_{i}$. As a consequence, based on the variables of the problem $y_{i}\in \left\{0,1\right\}$, the ORST scheme can be considered as a Binary Integer Problem (BIP).

The constraint presented in Equation (3C) is determined by the coordinator node, where matrix C represents the set of nodes that do not have an adequate communication link with the coordinator node. Each row of matrix C represents a node $x_{i}$ that does not communicate directly with the coordinator and each column represents a node that is able to hear this node. In this case, *d* will be equal to 1, in order to guarantee that at least one of these nodes will cooperate with node $x_{i}$.

**Figure 7:**
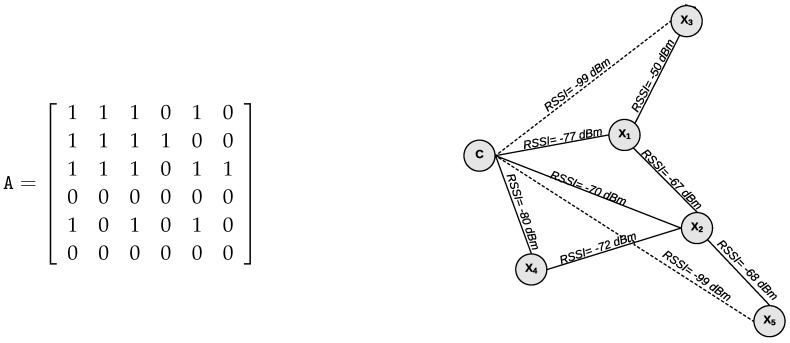
Network with the neighborhood of each node.

Figure 7 illustrates the operation of these constraints, where nodes are considered to be neighbors if RSSI ≥-87 dBm. In this case, the coordinator *C* (represented in row 1 of matrix A) received the list of neighbors of nodes $x_{1}$, $x_{2}$ e $x_{4}$. Thus, matrix A is equal to:

As the coordinator node did not receive the list of neighbors from nodes $x_{3}$ and $x_{5}$, rows 4 and 6 are equal to zero. Constraint (3b) is set up as follows: $x_{1}$: $y_{1}+y_{2}+y_{3}\geq 1$, $x_{2}$: $y_{1}+y_{2}+y_{4}+y_{5}\geq 1$ and $x_{4}$: $y_{2}+y_{4}\geq 1,$ where each $x_{i}$ is composed of the neighborhood of node $x_{i}$. This constraint ensures that each node will have at least one relay among its neighbors, that is, one or more binary variables $y_{i}$ must be equal to one ($y_{i}=1$). Constraint (3c) is formed by the nodes from which the coordinator did not receive the list of neighbors, that is, $x_{3}$ and $x_{5}$.

For each node $x_{i}$ that does not communicate with the coordinator, a constraint is set up with the binary variables of nodes $x_{j}$ that listen to node $x_{i}$, and one of these nodes must be relay. That is, the binary variable of this node must be equal to one ($y_{i}=1$). In this case, the list of nodes that listen to nodes $x_{3}$ and $x_{5}$ is formed only by, respectively, nodes $x_{1}$ and $x_{2}$, being: $x_{3}$: $y_{1}=1$ and $x_{5}$: $y_{2}=1$. The benefit function will prioritize the selection of nodes with smaller profit that respect the constraints. The relative weights of the parameters of the benefit function can be modified, according to the values of $\beta^{v},\beta^{e},\beta^{s}$ e $\beta^{H}$.

As previously mentioned, the ORST proposal represents a Binary Integer Problem. This type of problem can be solved by a Branch and Bound approach, which in the worst case runs on exponential time [43]. In the simulation assessment, a solver integrated to the simulation tool has been used to solve the formulated problem for all of the analyzed scenarios. The analysis of its execution time and the comparison with other solving methods are out of the scope of this paper.

Finally, in ORST, an adequate number of slots is reserved for the communication, enabling all nodes to transmit their messages, and also all relay nodes to retransmit their messages, at each period of the network. The proposed ORST relay selection scheme assumes that there is a limited number of relay nodes. Considering a maximum number of 100 nodes in the network, it is assumed that up to 40% of these nodes can act as relays. Therefore, a total of 140 slots should be reserved in the network for the transmission and retransmission of all messages.

### 3.4. Relay Updating Schemes

In this paper, two relay updating schemes have been investigated, the Periodic Relay Selection (PRS) and the Adaptive Relay Selection (ARS). The periodic relay selection scheme is an extension of preliminary work reported in [19], where the problem of high energy consumption has been corrected by adjusting the number of BIs that the nodes need to stay awake (described in Section 3.2). In the adaptive relay selection scheme, a new relay selection will be triggered based on the network success rate. That is, if the success rate is smaller, the relay selection will be more frequently triggered, if the success rate is higher, the relay selection will be performed with a lower frequency.

Periodic Relay Selection (PRS) — In the PRS scheme, the time interval between two consecutive relay selections $T_{IS}$ is fixed and independent of the current relay performance.

**Figure 8:**
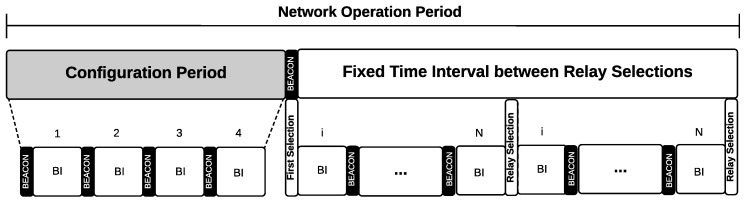
Operation of the periodic relay selection scheme.

The operation of the PRS scheme is illustrated in Figure 8, where the first four BIs (Beacon Intervals) characterize the configuration phase. At the end of this phase, the first relay selection will occur, the time interval for the subsequent selections being periodic.

In a network with a static topology, $T_{IS}$ may have a higher value, considering that the set of nodes remain in the same localization. On the other hand, when considering dynamic topology networks, lower values for $T_{IS}$ allow new relays to be selected sooner if any modification in the network occurs. Nevertheless, the proposed scheme does not take into account the performance of the current set of relays. That is, even if the network success rate is 100%, a new selection will be triggered every $T_{IS}$.

Adaptive Relay Selection (ARS) — The ARS scheme allows an adaptive selection of the relay nodes for networks with dynamic topology, where several nodes may join or leave the coverage area of the coordinator node. In the proposed scheme, the interval between two consecutive relay selections $T_{IS}$ is dynamically determined, according to the success rate of the network. The reason for considering the network’s success rate as a whole and not just the link between the cooperating nodes and the coordinator is that the communication of all these nodes with the coordinator can remain acceptable over time. However, with nodes joining and/or leaving the coverage area of the coordinator node, it may occur that other nodes require cooperation and the current set of relays nodes may no longer be adequate. Thus, if all messages successfully reach their destination, there is no need for a new relay selection. However, if the success rate decreases, it means that the current set of relay nodes is not meeting the communication requirements of the network and a new relay selection procedure must be triggered.

**Figure 9:**
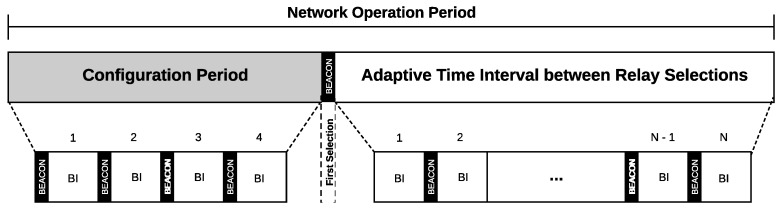
Operation of the adaptive relay selection scheme.

The operation of ARS is illustrated in Figure 9. When the network starts, as in the periodic scheme, a configuration phase of four BIs is also considered. At the end of the configuration phase, the first relay selection will occur, and the interval for the subsequent selections will be dynamically determined according to the success rate of the network.

Before sending a new beacon, the coordinator node checks the network success rate. If it has increased more than δ, the interval between selections ($T_{IS}$) is incremented by one BI, respecting an upper bound of 10 BIs. However, if the success rate has decreased more than δ, the $T_{IS}$ value is reduced by half of its current value, respecting a lower bound of 2 BIs. If the success rate remains δ-stable, the $T_{IS}$ value will be kept at its previous value. The maximum time interval between two consecutive selections has been set to 10 BIs to ensure the responsiveness of the network. These up and down selection rates were obtained by simulation using the OMNeT++/Castalia simulator.

After defining the $T_{IS}$ value, the coordinator node needs to inform the nodes about when the relay selection will be performed. Thus, the next two beacons will be used to implement the relay selection. Firstly, the coordinator informs the network nodes through the first beacon, then notifying them to listen to their neighbors. In this way, the nodes know that they need to listen and update their list of neighbors during this BI. In the next BI, each node will send its updated list of neighbors piggybacked with the data message. After receiving the message from the nodes containing the list of neighbors, the coordinator will select a new set of relay nodes.

## 4. Simulation Assessment

The network simulation tool OMNeT++ [20] and the WSN framework Castalia [44] were used to evaluate the proposed ORST scheme, with the PRS and ARS relay updating schemes. The open source Solve Library *lp_solve* [45] was used to implement the relay selection in ORST, solving the resulting optimization problem.

### 4.1. Characteristics of the Model Implementation

In Castalia, several extensions were added to the available IEEE 802.15.4 LLDN model, including the *aNumSuperframeSlots* parameter, the number of guaranteed time slot (GTS) slots, and the size of contention access period (CAP). This was necessary because Castalia still does not have a fully functional implementation of the LLDN communication mode.

The *aNumSuperframeSlots* parameter determines the size of the active portion of the superframe. The default value in the standard is 16. As previously mentioned, we consider 140 slots for the transmission and retransmission of messages. The superframe also considers the time corresponding to five time-slots during the CAP for node association, according to the IEEE 802.15.4 standard. This value of *aNumSuperframeSlots* parameter (145 slots) constrains the values used for both the *Beacon Interval* (BI) and the *Superframe Duration* (SD). These BI and SD values define the duty-cycle of the network, that is, its periodicity and the duration of its inactive period.

### 4.2. Simulation Settings

Five simulation scenarios were defined with 21, 41, 61, 81 and 101 nodes, one of the nodes being the Personal Area Network (PAN) coordinator. Nodes were randomly deployed in an area of 50 × 50 m${}^{2}$, with the PAN coordinator positioned in the center. The used channel model was the free space model without time-varying. Other simulation parameters are described in Table 2.

The simulation execution time was set to 450 seconds, during which the coordinator is able to send up to 50 *beacons*. The radio model used was CC2420, which is compliant with the IEEE 802.15.4 PHY Standard. For the PRS updating scheme, the interval between relay selections ($T_{IS}$) was defined to four BIs. This value was obtained by simulation using the OMNeT++/Castalia simulator. Thus, in the case of a modification of the network, a new selection of cooperators will be quickly initiated. To reduce the statistical bias, each simulation was performed 60 times with a confidence interval of 95%.

The simulations were performed considering two modes of operation. Firstly, a static topology has been considered, where all nodes remain connected to the network until the end of the simulation. Then, a dynamic topology was also considered, where only 50% of nodes were associated with the network at time zero and the remainder were subsequently associated in groups of 5 by 5 nodes. The first group at time instant 50 s and then all the other groups every 30 s. Considering the scenario with the highest number of nodes (100 nodes), after 320 s, all nodes were associated. Later, from the time instant 320 s of simulation, 20% of the nodes of the network randomly left the coverage of the coordinator node. This leaving operation was performed in groups of four nodes, every 10 s of simulation. Finally, all nodes again joined the network, in the same order they have left (groups of 4 in 4), from the time instant 350 s of simulation, respecting an interval of 10 s for each group, except for the case of the network with 100 nodes, where only 10% of the outgoing nodes returned.

The dynamic topology mode was designed to force the list of neighbors to undergo multiple changes during the simulation time, in order to assess the reliability of the relay selection procedure.

### 4.3. Compared Techniques

To validate the relevance/pertinence of the proposed PRS and ARS relay updating schemes, in addition to comparing the two techniques among themselves, their performance was also compared to three state-of-the-art techniques: Completely Random selection [22], Random selection Around the Coordinator [21] and Opportunistic selection [7]. This subsection briefly describes these three state-of-the-art relay selection techniques.

Completely Random (CR) — A totally random technique [22] was considered for the selection of the relay nodes, without considering the quality of the communication link with the coordinator. To determine the number of relay nodes, it must be verified how many nodes are associated with the network. If the number of associated nodes is smaller than the maximum number of relays (n_r) (defined in Section 3), then the maximum number of relays is the number of associated nodes. Otherwise, a random number of relays between 1 and the maximum number of relays is selected (Equation (4)):

(4) numCoop=random[1,n_r].

In this case, the set of numCoop relay nodes is randomly selected.

Random Around the Coordinator (RAC) — The random selection technique is a simple technique [21] that randomly selects relay nodes that are closely located around the coordinator, by using the RSSI metric as the selection criterium. For the selection of cooperating nodes, the signal strength, between the node and the PAN coordinator was lower bounded to -87 dBm.

At each selection, the maximum number of relays (n_r) is determined by Equation (5):

(5) $$\begin{array}{c} n\_r=min(min(n\_cl,n\_ncl),n\_cm), \end{array}$$

where:

- n_cl is the number of nodes that the coordinator is able to listen, considering the RSSI lower bound of -87 dBm;
- n_ncl is the number of nodes that the coordinator is not able to hear;
- n_cm is the maximum number of relay nodes that can be selected; this upper bound was set to 40, as previously mentioned in Section 3.

Equation (5) determines the maximum number of relay nodes, according to the smallest value between n_cl and n_ncl to prevent a large number of relays from being selected. The second part of the equation ensures that if the two cited variables are greater than 40, then this upper bound value is selected. In this way, the number of selected relays will always be a value between 1 and n_r (Equation (4)).

Finally, the set of numCoop relay nodes is randomly selected among nodes that have a good communication ratio with the coordinator.

Opportunistic — Finally, the technique proposed by Valle et al. [7] presents an opportunistic selection of relay nodes. In this technique, the number of relay nodes is determined according to the network error rate. As the error rate can quickly fluctuate over time, an exponentially weighted moving average is used, which keeps the “memory” of the last instances. The calculation of the relative weights between the last measured instance is done using two constants: α and β.

The evaluation of the new set of relay nodes is executed at each relay selection. The upper bound for the number of relays is given by the number of potential cooperative nodes $\left(n_{p}\right)$. In this evaluation, the proposed technique considers the number of previous unsuccessfully transmitted messages. It involves the estimation of the number of message losses $\left(E_{L}\right)$ and its standard deviation $\left(D_{L}\right)$:

(6) $$numCoop=min(n_{p},\left(\delta \times E_{L}\right)+D_{L}).$$

The estimated number of message losses $\left(E_{L}\right)$ is an exponential moving average based on a weighted combination of the previous value of $E_{L}$ and the new value of $S_{L}$, which is the number of messages that have not been successfully delivered in the previous beacon interval:

(7) $$E_{L}=\left(1-\alpha \right)\times E_{L}+\alpha \times S_{L}.$$

The standard deviation $\left(D_{L}\right)$ is an estimation of how much $S_{L}$ typically deviates from $E_{L}$:

(8) $$D_{L}=\left(1-\beta \right)\times D_{L}+\beta \times \left|S_{L}-E_{L}\right|.$$

To determine the best set of relays, the authors use a predefined communication quality index $\left(Q_{i}\right)$. The mean value of these two estimators is used for the $Q_{i}$ calculation: the success rate $\left(H_{i}\right)$ and the normalized link quality indicator $\left(L_{i}\right)$. The $L_{i}$ value is used mainly because it has a good correlation with the success rate. Therefore, for each node *i*, its communication quality index $\left(Q_{i}\right)$ is evaluated at the coordinator as:

(9) $$Q_{i}=\frac{H_{i}+L_{i}}{2}.$$

The $H_{i}$ value is evaluated as:

(10) $$H_{i}=\left(1-\alpha \right)\times H_{i}+\alpha \times S_{R},$$

where $S_{R}=1-S_{L}$.

The values of δ, α and β constants for Equations (6)–(8) and (10) were tuned, and the selected value was 2 for δ and 0.2 for both α and β [7]. The coordinator node maintains an ordered list of $Q_{i}$ values and the set of numCoop nodes with higher $Q_{i}$ value will be elected as relays.

### 4.4. Simulation Assessment

The simulation assessment considered the following metrics: success rate, number of cooperation per node, energy consumption, number of relay selections during the system’s runtime and the percentage of duplicate (useless) messages. The success rate represents the ratio between the number of sent messages and the number of messages that actually reach the coordinator. This metric considers messages transmitted in both the transmission attempt and the retransmission attempts performed by relayers. The number of cooperations represents the average number of cooperations performed per node, i.e., it is based on the number of retransmission messages sent by each relay node. Energy consumption represents the average amount of energy spent by each node, obtained through the resource management module available in Castalia framework. The average number of relay selections represents the average number of times a new relay selection was triggered during the simulation time. Finally, the percentage of duplicate (useless) messages represents the percentage of cooperation’s messages that were not used, i.e., all messages that the relay node listened to and inserted in the cooperation message that had already arrived with success to the coordinator.

**Figure 10:**
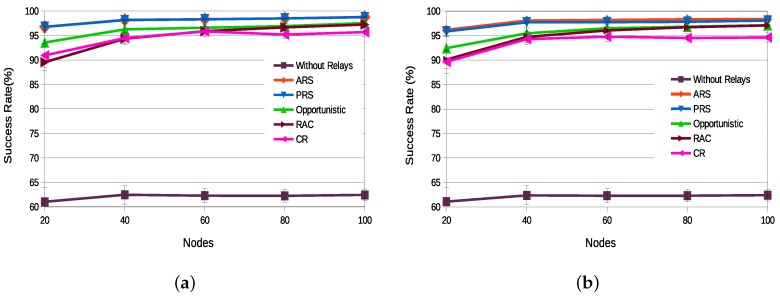
Success Rate—static topology (**a**) vs. dynamic topology (**b**).

Figure 10a,b illustrate the success rate of all assessed relay selection techniques for static and dynamic network topologies, respectively. Both figures clearly highlight the importance of the relay retransmission techniques, as when retransmission techniques are not used (“Without Relays”), the obtained success rate is smaller than 65%. It is worth noting that, despite the good performance of state-of-the-art techniques, the proposed selection schemes ARS and PRS present better performance, with success rate above 95%, independently of the number of nodes and topology. For the static topology, ARS and PRS have basically the same probability of successful transmissions. For the dynamic topology network, where nodes may join/leave the network, the ARS updating scheme has a slightly better performance than the PRS. Among the state-of-the-art techniques, the Opportunistic scheme presented the best success rate results, and the Completely Random (CR) scheme presented the worst results.

**Figure 11:**
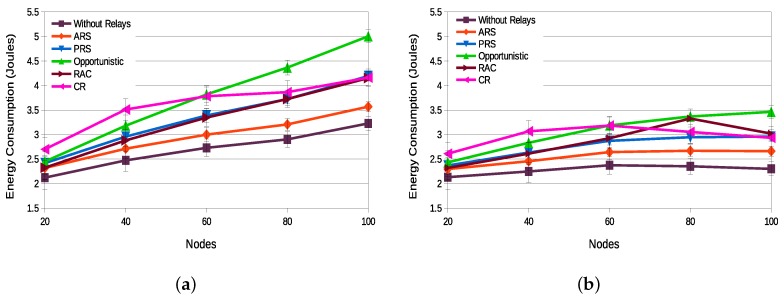
Energy consumption—static topology (**a**) vs. dynamic topology (**b**).

Figure 11a,b present the energy consumption in the network with static and dynamic topology scenarios, respectively. For both scenarios, the ARS technique presents the lower energy consumption, followed by the PRS and RAC techniques in the static topology scenario and the PRS technique in the dynamic topology scenario. The ARS technique presents an energy consumption lower than PRS because nodes do not need to listen and update the list of neighbors so frequently, it only being necessary to update the list of neighbors whenever a new relay selection is really necessary.

**Figure 12:**
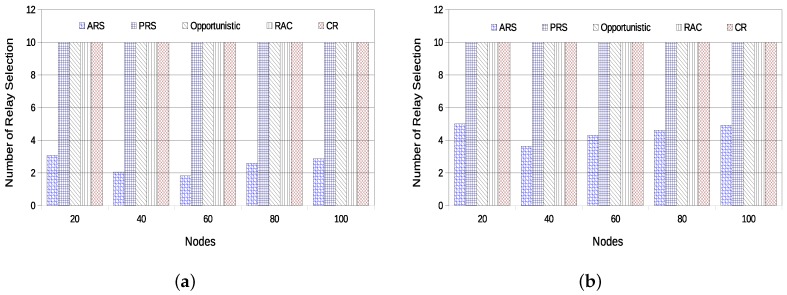
Average Number of Relay Selection—static topology (**a**) vs. dynamic topology (**b**).

As the previous results have already shown the advantage of using cooperative diversity techniques, the next results discuss only metrics related to cooperation techniques. Figure 12a,b present the average number of relay selection operations in the network with static topology and dynamic topologies, respectively. It is possible to verify that the average number of relay selections made by the ARS technique in the static scenario is much smaller than in the dynamic scenario. This is due to the fact that the success rate of the network is just slightly changing during the simulation time for the static topology. On the other hand, for the dynamic topology scenario, the network suffers a significant number of modifications, imposing a higher rate for the relay selections.

**Figure 13:**
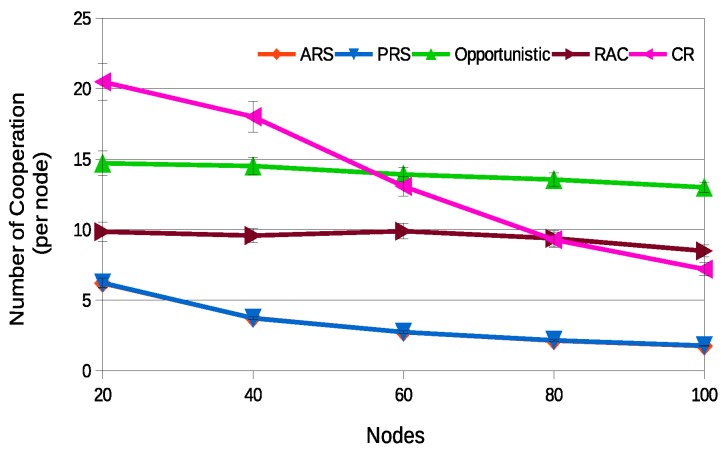
Average number of cooperation exchanges for a dynamic scenario.

As dynamic topology scenarios, where nodes can dynamically join and leave the network, are more challenging for the retransmission techniques, for the sake of simplicity, hereafter in this paper, only the results for the dynamic topology scenarios will be represented. Figure 13 illustrates the average number of cooperation per node. As expected, the ARS and PRS have the smaller number of cooperations. This behavior is a direct consequence of the smaller number of selected relays, due to the employed optimization technique. This metric associated with the success rate allows for verifying how many cooperations per node were required to reach the adequate level of success rate. For instance, in the ARS and the PRS techniques when the number of nodes is 20, the average number of cooperations performed per node is about 6 reaching a success rate about 96%, while, in the CR technique, the average number of cooperations performed per node is about 20 and the success rate is about 89%.

The CR technique is the one that presents the highest average number of cooperations per node in scenarios with fewer nodes (less than 60) because the probability that the same nodes will be selected as a relay is greater.

**Figure 14:**
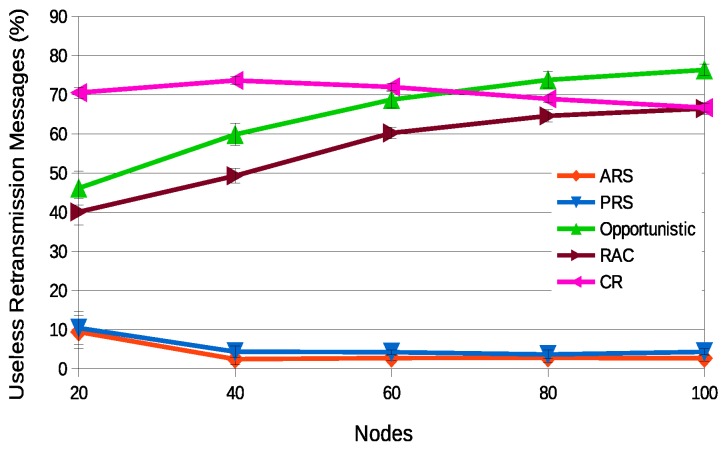
Percentage of useless retransmission messages for a dynamic scenario.

Figure 14 presents the percentage of useless retransmission messages, where ARS is the relay selection technique that presents the smallest value, followed by PRS. That is, in both schemes, the set of relay nodes has been optimized by the proposed optimization procedure. Other techniques have more than 50% of useless retransmission messages, i.e., these messages were already received in transmissions or retransmissions performed by other nodes. For the case of the Opportunistic technique, the justification is that the number of relays is determined by the success rate of the network. Therefore, whenever the success rate decreases, a higher number of nodes will be selected. For the case of the Completely Random (CR) and Random Around the Coordinator (RAC) techniques, it occurs because they do not consider any criteria to determine how many nodes will be selected as relays nodes, selecting an unnecessary number of relay nodes. Again, among the state-of-the-art techniques, the RAC scheme presented the best results.

**Figure 15:**
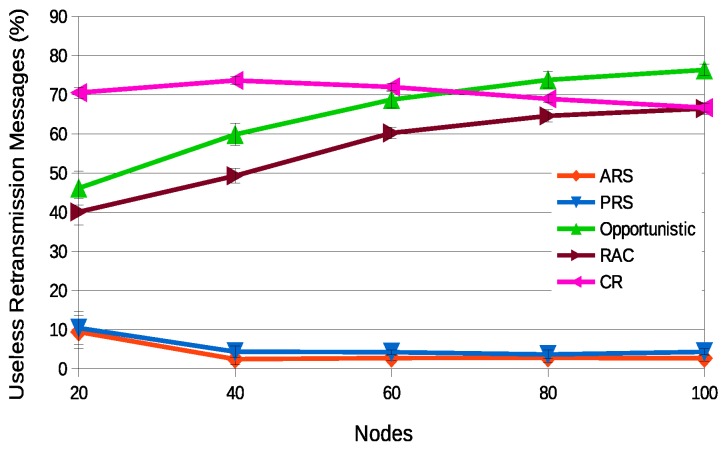
Correlation between energy consumption and useless retransmission messages.

Finally, Figure 15 illustrates a correlation between energy consumption and useless retransmission messages of the two schemes proposed in this work (ARS and PRS) and RAC, which was the-state-of-the-art technique that obtained the best performance. It is possible to observe that ARS is the technique that presents the lowest energy consumption, being one of the main reasons the transmission of the smallest number of useless messages.

## 5. Conclusions

Smart objects with communicating actuating capabilities are becoming more common, bringing the IoT paradigm closer, where sensors and actuators are completely integrated into the environment and communicate transparently. The use of WSNs have been pointed out as a promising technology for the IoT paradigm. However, and due to the inherently unreliable wireless communication medium, new communication schemes are needed to increase WSNs’ communication reliability.

Cooperative diversity techniques can be used to improve the reliability of WSN communications. Nonetheless, an important step for the use of these techniques is to perform an adequate relay selection. This paper focuses on the adequate selection of relay nodes. It proposes the use of the ORST scheme, whose target is to adequately select relay nodes without generating overheads or excessive energy consumption.

The proposed ORST relay selection scheme was applied using two relay updating schemes: PRS and ARS. Aiming to investigate which is more adequate to improve the reliability of WSN, the following metrics were considered: success rate, average number of cooperation exchanges per node, percentage of duplicate (useless) messages, energy consumption and average number of relay selection.

Both PRS and ARS schemes were assessed by simulation against other three state-of-the-art relay selection techniques. The performed simulation assessment highlighted that ORST demonstrates a significantly improved reliability behavior for both updating schemes: PRS and ARS. The proposed ARS relay selection technique outperformed the other state-of-the-art techniques, increasing the message transfer success rate, decreasing the average number of cooperation exchanges per node, and presenting the smallest percentage of useless retransmission message and a lower energy consumption. That is, the use of an optimized relay selection technique is able to more efficiently select the set of relay nodes.

As future work, we intend to assess the implementation feasibility of the proposed scheme using available COTS (commercial off-the-shelf) WSN nodes. In this case, it will be necessary to evaluate and to optimize the resolution method implemented to solve the generated optimization problem, as typical WSN nodes have scarce available resources—mainly related to reduced footprint memory and processing capabilities.

## Figures and Tables

**Table 1:** Relay selection techniques.

State-of-the-Art	Category	Periodic or Adaptive	Exchange of Additional Messages
Liu et al. [26]	Link Quality	P	
Marchenko et al. [16]	Link Quality	P/A	√
Li et al. [13]	Link Quality	P	√
Ferdouse and Anpalagan [14]	Link Quality	P	
Andre et al. [27]	Link Quality	A	√
Valle et al. [7]	Link Quality	P	
Etezadi et al. [21]	Link Quality and Neighborhood	P	√
Alkhayyat, Gazi and Sadkhan [15]	Link Quality and Neighborhood	P	√
Brante et al. [28]	Link Quality and Energy	P	
Ahmed, Razzaque and Hong [29]	Link Quality and Energy	P	√
Pham and Kim [17]	Link Quality and Energy	P	√
Cheikh, Simpson and Sun [30]	Link Quality and Energy	P	√
Gokturk and Gurbuz [31]	Link Quality and Data Rate	P	
Ouyang et al. [32]	Link Quality and Data Rate	P	√
Willing and Uhlemann [22]	Random Relay Selection	P	
Proposed Technique	Multi Parameters	P/A	

**Table 2:** Simulation setting.

Parameters	Values	Parameters	Values
Node distribution	Random with coordinator in center	Beacon Order (BO)	6
Radio	CC2420	Superframe Order (SO)	4
MAC layer	IEEE 802.15.4	$\beta^{n}$	0.5
Number of superframe slots	145 (5 are used by the CAP)	$\beta^{e}$	1.5
Data rate	250 kbps	$\beta^{s}$	1.0
Initial energy per nodo	18,720 J	$\beta^{H}$	1.5
TxOutputPower	0 dBm	$T_{IS}$	4 (for PRS)
